# Employees in the municipal healthcare service experiences with participation in quality improvement collaboratives and the use of the Quality Improvement Model: a qualitative study

**DOI:** 10.1186/s12913-026-14004-4

**Published:** 2026-01-16

**Authors:** Kari Blindheim, Helen Berg, Lindis Kathrine Helberget

**Affiliations:** Department of Health Sciences Ålesund, Norwegian University of Science and Technology, Ålesund, Norway

**Keywords:** Focus group, Healthcare professionals, Municipal health service, Quality improvement, Quality improvement collaborative, Quality Improvement Model, Quality improvement work

## Abstract

**Background:**

Quality improvement is a continuous process, and quality improvement collaboratives are frequently used worldwide to promote quality improvement. A Quality Improvement Model is recommended for performing quality improvement work systematically to ensure both quality and patient safety in healthcare services. The aim of this study was to gain a deeper understanding of municipal healthcare service employees’ use of the enhanced first phase of the Quality Improvement Model and to explore their experiences of participation in digital quality improvement collaboratives.

**Methods:**

A qualitative design with focus group interviews was used. A semistructured interview guide was developed by the authors prior to the focus group interviews with the goal of obtaining relevant data for this study. Reflexive thematic analysis was used to analyse the focus group interviews.

**Results:**

Three themes were revealed by the analysis: learning in community with others in a digital quality improvement collaborative; it is important to use the Quality Improvement Model properly; and the establishment of a culture that is conducive to quality improvement work is important. These themes highlight the necessity of ensuring that both managers and employees are familiar with the Quality Improvement Model used in quality improvement projects and understanding the importance of dedicating sufficient time to the preparation phase. Digital quality improvement collaboratives sessions and interim process guidance were key to sustaining momentum and driving progress in the quality improvement work.

**Conclusion:**

The Quality Improvement Model is suitable for quality improvement work; furthermore, by using the model, participants were able to remain in the preparation phase longer, thereby refining their quality improvement work and making the project more robust. The digital quality improvement collaborative sessions and the process guidance provided between the sessions were important with respect to the progress of quality improvement work. Leadership anchoring and involvement are essential with respect to efforts to encourage employees to participate in quality improvement work and implement the changes required by such work.

## Background

Quality improvement (QI) is a continuous cyclic process [1], and QI work refers to changes in work practices or work organizations that result in better patient outcomes [2].The efforts dedicated to QI work at a workplace are influenced by the presence or absence of a culture that is conducive to QI at both the organizational and operational levels [1, 3]. Much of the QI work has been dominated by North American methodologies and in this study, we offer insights into the Quality Improvement Model [4] used in quality improvement collaboratives (QICs). QICs are frequently used worldwide to promote QI work, disseminate knowledge to healthcare professionals, and support QI work in healthcare units [5]. A QIC is a composite team that consists of employees who work in a structured manner under professional guidance with the goal of improving specific aspects of healthcare services [5].

**Fig. 1:**
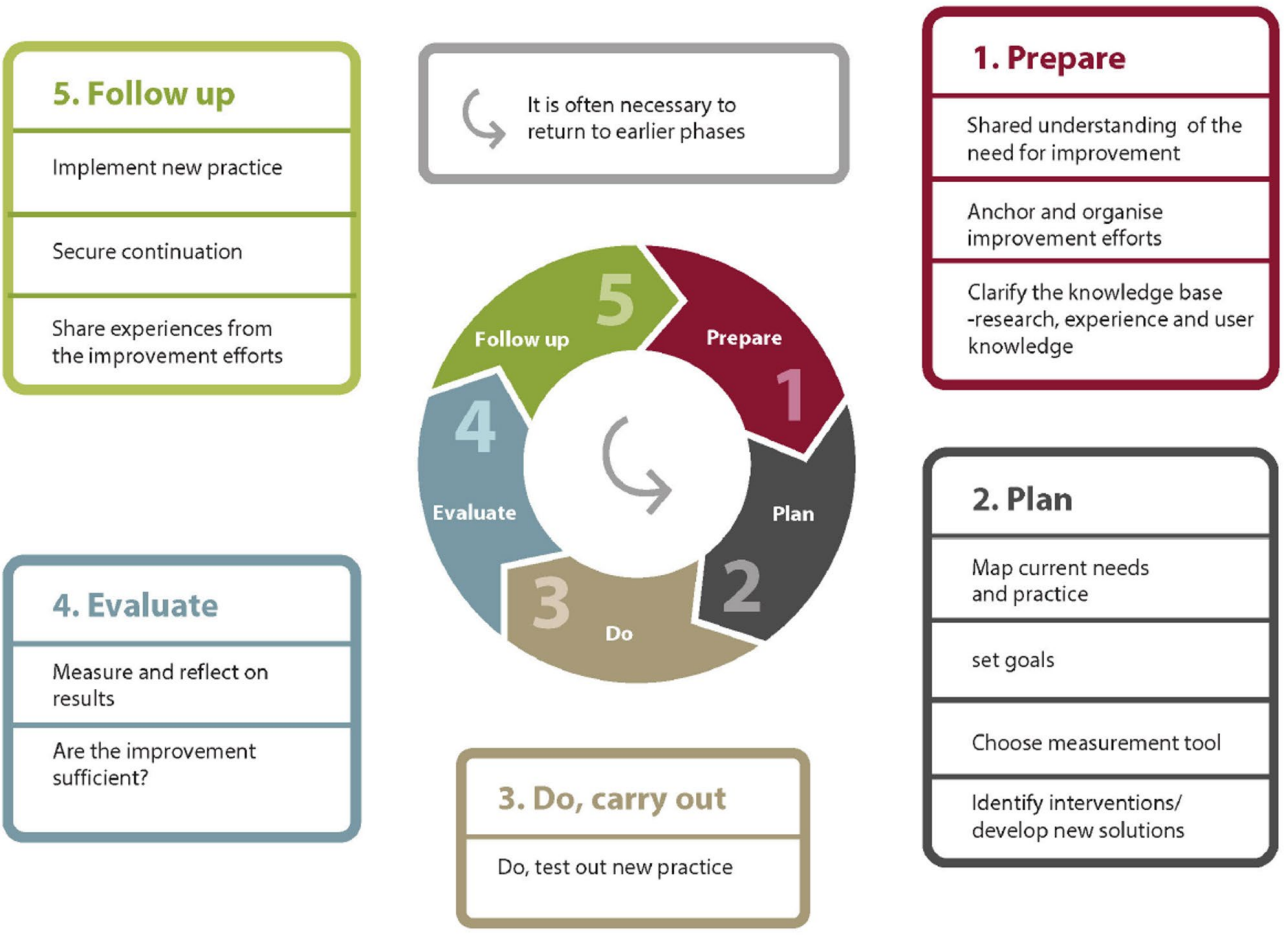
The Quality Improvement Model [4]

Research has shown that providing sufficient and appropriate external support to the teams included in the QIC is important to enable them to function well and achieve successful organizational change [6–8]. Health care professionals who participate in a QIC appreciated the support and methodological guidance they received from QI specialists in their QI work, and they considered the QIC to be a driver of the QI methodology [9]. Participation in QICs can positively influence the knowledge, attitudes, and problem-solving abilities of healthcare professionals [7], and they have a higher degree of job satisfaction, lower absenteeism and a higher level of patient care [10]. Leadership engagement, an organizational culture that promotes QI work, and the allocation of resources are important with respect to efforts to engage employees and ensure the success of QICs and QI work [7]. However, no linear relationships have been shown between the design of the QIC and sustained improvement in outcomes; nonetheless, QI work that focuses on increased patient follow-up or standard care programs can lead to long-lasting improvements in outcomes [11, 12]. Additionally, a study highlighted the potential to save costs in both acute and chronic healthcare systems by using QICs in QI work [13]. However, the existing QIC literature has little attention to this particular QI model (Fig. 1) used in a QIC.

### The Quality Improvement Model

The Quality Improvement Model developed by the Knowledge Centre for Health Services in Norway [4, 14] is expanded on the traditional Plan-Do-Study-Act (PDSA) cycle [15], by adding a preparation phase before the planning phase. This new preparation phase in the Quality Improvement Model focuses on a shared understanding of the need for improvement, thereby anchoring leadership and emphasizing thorough planning and clarification of the knowledge base. Second, an extended follow-up phase focuses on implementing new practices, ensuring continuation, and disseminating improvements [16]. These additions were all important areas requested in the PDSA cycle to ensure successful QI work [17].

Using a QI model to guide the performance of a QI work can ensure better execution [18], determining the particular QI model that is selected for a given project is not important, as long as the model is appropriate for its purpose [19]. Nevertheless, the task of measuring the effect of QI projects has been proven to be difficult [20].

To the best of our knowledge, research on the use of this specific Quality Improvement Model in digitally QICs is lacking. This study may provide insights into whether the enhanced first phase can contribute to strengthening QI work, a need that emphasized in previous research. In addition, experiences of participation in digital QICs may provide knowledge of significance for the design of future QICs. This may have implications beyond the Norwegian context. The aim of this study was to gain a deeper understanding of municipal healthcare service employees’ use of the enhanced first phase of the Quality Improvement Model and to explore their experiences of participation in digital quality improvement collaboratives. The research question was: How do healthcare professionals in municipal healthcare services experience their participation in QICs and the use of the Quality Improvement Model in practice?

## Materials and methods

### Design

This study had a qualitative design, which can be used to gain a deeper understanding of healthcare professionals’ experiences [21]. Data were collected through focus group interviews. When the aim is to illuminate attitudes and experiences, focus group interviews are considered as an appropriate method [22]. Compared with individual interviews, focus group interviews can bring forwards more spontaneous, expressive and emotional aspects [21].

### Setting

The QIC was caried out in the municipal healthcare services in Norway, including nursing homes and home care services which constitute the highest level of municipal health care services. The QIC in this study is a refinement of the Collaborative Model of the Institute of Healthcare Improvement (IHI) [23]. In this service, people with different competencies work, from care assistants to specialist nurses. From 2021 to 2022, two QICs were established with 22 teams comprising 136 employees from 10 municipalities in central Norway. This was in the middle of the COVID-19 pandemic, thus the QIC had to be carried out digitally. The QICs were led by the Centre for Development of Institutional and Home Care Services (USHT). Each QIC included four digital sessions, and in addition, each team received guidance on group work between the sessions from a supervisor affiliated with the USHT staff. The supervisors received training by experts from the Centre for Care Research (SOF) and the Norwegian Directorate of Health.

### Recruitment and participants

All the participants in the QIC were invited to participate in the study by the USHT. Those who accepted the invitation are included in the study. The inclusion criterion was participation in one of the two QICs. The recruitment process was led by the head of the USHT and involved distributing information regarding the study to all participants in the QICs. Individuals who agreed to participate in this study made direct contact with the first author. Ten participants agreed to participate in this study; all of these participants were women whose tenure in the municipal health service ranged from 1 to 23 years, with an average of 10 years. The first author divided the participants into three focus groups (Table 1). The participants from focus groups two and three were from the same QIC teams.

**Table 1:** The participants in the focus groups

Participants	Focus group 1	Focus group 2	Focus group 3
Caregiver coordinators Sections leader in nursing home Specialist nurse in home care services	1 1 1		1
Specialist nurse in a nursing home Nurse with a master’s degree in a nursing home Manager in a nursing home		1 2 1	
Dietitian Healthcare assistant in a nursing home			1 1

### Data collection

All three focus group interviews were conducted in 2022, either in a meeting room at a health care centre or at the university. A semistructured interview guide (Table 2) was developed by the authors prior to the focus group interviews to obtain relevant data to support this study. The themes contained in the interview guide included experiences with participation in QICs, whether participants experienced that the intervention involving QI work in which they had participated resulted in improvements to health and care services, and participants’ perceptions of the use of the Quality Improvement Model. The focus group interviews were conducted by the first author and moderated by the last author; they lasted between 62 and 70 min each. The focus group interviews were recorded with an audio recorder. and transcribed verbatim.

**Table 2:** Interview guide

• Could you describe your experiences of participating in QIC
• Can you say something about your experiences of using QIC for your QI work?
• How do you experience the professional support and guidance by USHT during and between the QIC session?
• Could you share your experience with the training in the Quality Improvement Model?
• Could you share your experience with the application of the Quality Improvement Model?
• What do you consider to be the most significant change in practice compared to the period prior to participating in the QIC?
• What factors do you believe are crucial for maintaining such changes over time?
• What is required for the knowledge and competence you have acquired to be transferable to other QI project?
• Do you have any final reflection, thoughts, or comment you would like to share at the conclusion of this interview?

### Ethical considerations

The participants received both oral and written information regarding the project and signed a consent form prior to their participation in this study. They were informed that their participation in this research was voluntary and that they could withdraw from the study without suffering any consequences. The interview data were stored in accordance with Norwegian guidelines and regulations for research [24], and all the data were anonymized during the transcription process [21]. The study was assessed by the Data Protection Services for Research at SIKT and approved under reference number 692,337 [25].

### Data analysis

The focus group interviews were transcribed verbatim and analysed by reflexive thematic analysis in a six -phase approach by Braun and Clarke [26, 27]. The first and last authors read and reread the interview transcripts and coded the entire dataset with the aim of identifying data that were meaningful to the aim of the study individually and subsequently convening to compare the codes. The first author then organized the codes and identified potential themes before all the authors met again and developed a digital mind map that provided an overview for subsequent analysis. In this process, the authors discussed the preliminary themes with reference to the quotes and codes. The themes were further reviewed and refined by the authors in an iterative process (Table 3) until three themes and eight subthemes were identified and given informative names (Table 4). The first author wrote the first draft of the results. The thematic analysis was conducted inductively, with codes and themes developed from the data. Reflexivity was maintained through ongoing reflection and discussion among the authors, accompanied by a continuous awareness of our preunderstanding [26].

**Table 3:** Example from the analysis process

Theme	Subtheme	Code	Quote
It is important to use the Quality Improvement Model properly	The dedicate significant time during the preparation phase The model’s provisions of structure and support	The participants were impatient during the preparation phase and wanted to move on to the testing step The model is useful at the start of a project; they know how to proceed	We often received feedback indicating that we were moving too quickly; it was probably because we were more focused on the actions themselves than on the project (7)(P7) The idea of stopping to evaluate and correct, not just pushing forwards; you have to go through all the steps, which makes it highly applicable (6)(P6)

## Results

The analysis revealed three themes that affected the participants’ experiences with QI work on the basis of the Quality Improvement Model as well as their participation in a digital QIC: learning in communities with others in digital QICs; it is important to use The Quality Improvement Model properly; and the establishment of a culture that is conducive to QI work is important.

Details are presented below under the headings of these three themes (Table 4).

**Table 4:** Overview of themes and subthemes

Themes	Subthemes
Learning in community with others in digital QICs	• Sharing experiences with the goal of increasing knowledge • The provision of process guidance between sessions can help maintain participants’ drive • Challenging digital sessions
It is important to use the Quality Improvement Model properly	• The dedication of significant time during the preparation phase • The model’s provision of structure and support
The establishment of a culture that is conducive to QI work is important	• The importance of leadership anchoring • Knowledge of QI work can improve individuals’ understanding • Challenges in efforts to maintain participants’ motivation over time

### Learning in community with others in digital QICs

Although the participants identified the digital nature of the meetings as challenging, they said that the sharing of experiences during sessions was educational and that the guidance between sessions helped them continue to progress in terms of their own QI work.

### *Sharing experiences with the goal of increasing knowledge*

Many participants said that exchanging experiences during QIC sessions had broadened their understanding of their professional knowledge and elicited a sense of community, which they missed when they made important decisions without discussing them with others:

*We often feel like we’re in our own little bubble*,* making it hard to see beyond ourselves. Being able to share experiences*,* thoughts*,* frustrations*,* and challenges means that we’re not alone*,* as there are many others there* (P2).

After exchanging experiences during the QIC sessions, the participants felt that they had obtained insights from others who had faced similar challenges. They viewed this exchange of experiences as both meaningful and resource saving. One participant expressed this point as follows:

*I was looking to see if they had something we did not have that we could acquire or something they could offer us as a “company”. It is exciting to hear how others do things* (P6).

Some participants expressed scepticism towards the numerous “sunshine stories” that were shared during the QIC sessions. These participants found these stories difficult to believe in light of their own experiences with the challenges associated with QI work. They stated that the challenges associated with QI work were not adequately addressed during the QIC sessions.

### *The provision of process guidance between sessions can help maintain participants’ drive*

The process guidance provided between the QIC sessions was described by the participants as crucial with respect to their understanding of the subsequent phase and their efforts to make progress in their various forms of QI work. The participants noted that despite the diversity of topics across the different teams, the supervisors were well prepared and familiarized themselves with the topics before beginning the supervision process. One participant discussed the guidance that their team had received:

*They are good at encouraging us; they do not immediately clash with us and criticize us. Instead*,* they are careful and suggest that we might try things differently. They have a very nice way of approaching us*,* which is encouraging* (P4).

The participants said that the guidance provided between the QIC sessions helped them make progress when they encountered obstacles in their QI work and that the supervisors consistently offered them positive feedback. Additionally, the participants mentioned that the submission deadlines and the requirements for teams to meet between sessions were beneficial with respect to such progress. With respect to the deadlines, one participant made the following statement:

*It was very helpful to have deadlines; they motivated us to deliver*,* complete*,* and work on it because we have a job where a thousand things are happening*,* so I appreciated that* (P6).

Another participant noted that the clear model, the established meeting points, and the deadlines made them feel assured and comfortable and suited their team well.

### *Challenging digital sessions*

Some participants mentioned that the digital sessions, which were hosted via the Microsoft Teams platform, made it challenging for them to absorb all the information shared during these sessions, and they mentioned that the physical presence of a lecturer in the room would have been more beneficial. Several participants highlighted experiencing a sense of insecurity regarding the possibility of speaking up during the QIC on Microsoft Teams, primarily because they were unfamiliar with the other participants. As one participant noted:

*It’s about the dynamics when there are so many people on Teams. There were many people from other parts of the county*,* so we did not know them at all. Speaking up is much harder on Teams*,* especially in large groups. You feel that what you say must be very important before you speak up*,* which isn’t easy* (P1).

Several of the participants claimed that if only one physical QIC session had been held at the beginning of the process, subsequent meetings on Microsoft Teams would have been easier for them. Some participants also found it difficult to maintain focus during the Microsoft Teams sessions since it was easy to multitask in this context:

*It was easy to make a phone call*,* step out*,* or work on something else on the side* (P6).

The participants expressed that they appreciated being divided into smaller groups in breakout rooms on Microsoft Teams, as these groups facilitated easier communication.

### It is important to use the Quality Improvement Model properly

The participants stated that thoroughness during the preparation phase of the Quality Improvement Model was beneficial. They also noted that the model provided them with structure and support in the context of QI work.

### *The dedication of significant time during the preparation phase*

Several participants had previously performed various types of QI work and had experience with the PDSA cycle. However, some participants found it unusual to dedicate so much time to the preparation phase as systematically as was the case in the QIC supported by the Quality Improvement Model. All the participants mentioned that in their busy everyday lives, it was easy for them to overlook the preparation phases of the model. However, they all recognized these phases as crucial with respect to QI work. One participant discussed this topic as follows:

*It’s about staying in the process. One might want to jump quickly to solutions*,* but I found standing firm and working through it systematically to be very beneficial* (P1).

Some participants mentioned that the process guidance provided between the QIC sessions prompted several teams to take a step back and revisit important aspects, such as by clarifying the knowledge base, leadership anchoring the work, and refining their understanding of the issue at hand. One participant expressed this point as follows:

*We often received feedback indicating that we were progressing too quickly; this was probably because we were more focused on the actions themselves than on the overall project* (P7).

Several participants described that “to be in the thick of it”—by avoiding rushing ahead—could enable them to uncover what initially seemed to be the problem, which was occasionally identified as a completely different challenge, by mapping their current practice.

### *The model’s provision of structure and support*

The participants described that the Quality Improvement Model provided them with a structured approach and support for their QI work. Although the model was comprehensive, they found it to be intuitive and easy to understand. The first steps included in the model were particularly appreciated, as noted by one participant:

*I feel that the initial parts are very useful when starting a new improvement project. Now I know where to begin and how to get started* (P3).

Several of the participants mentioned that a great deal of information regarding the model was available on the Norwegian Institute of Public Health website, which could be helpful if participants encountered difficulties or if the work began to stagnate. The participants emphasized the model’s usefulness for both small and large QI projects. They noted that the importance of pausing to evaluate whether an improvement had occurred became evident, especially since such recognition would highlight the possibility of adjusting their work if doing so became necessary. One participant made the following comment:

*The idea of stopping to evaluate and correct*,* not just pushing forwards; you have to go through all the steps*,* which makes the process highly applicable* (P6).

Moreover, the participants claimed that one challenge associated with QI work involves identifying high-quality indicators that can demonstrate whether quality has improved:

*Getting us to think about what we should measure and how we can do it and conducting a thorough investigation before diving in would be smart. It would be more enjoyable to see the results and obtain clear outcomes. I would have liked that. Measuring is useful because it shows how things are progressing* (P6).

All the participants expressed that the model made the process visible and provided them with support with respect to their QI work.

### The establishment of a culture that is conducive to QI work is important

Leadership anchoring was identified as central to QI work by the participants; furthermore, all employees understood the importance of QI work and were motivated since this process was continuous.

### *The importance of leadership anchoring*

The participants highlighted the importance of managerial support for QI work. Many participants appreciated the composition of teams that included both management and employees from various organizational levels. The participants highlighted the need for active managerial involvement in QI work and emphasized that continuous improvement work was a shared responsibility. While most participants said that it was not difficult to obtain management support, some faced challenges. One participant expressed this point as follows:

*It is about leadership anchoring; we struggled to get it across in the leadership group* (P10).

All the participants said that if management were not involved, it would be difficult to perform and implement QI work. One participant shared their experiences with the leadership anchoring of QI work as follows:

*It’s an important point you have there about leadership anchoring; all change efforts must be rooted in the leadership. There’s little that can be achieved if it comes from the bottom; you need goodwill from the leadership* (P8).

### *Knowledge of QI work can improve individuals’ understanding*

The participants expressed their agreement with the claim that all their colleagues needed to have wide knowledge of QI work and understand that QI work is a continuous process that often entails changes that could be demanding for participants. Moreover, the participants claimed that this understanding was essential with respect to ensuring that all employees engaged in the change process and followed through on the decisions made in this context. One participant expressed this point as follows:

*I think that what is important is ensuring that everyone understands its purpose [QI work]. When people understand “why” and “what”*,* they become more engaged. To avoid going over someone’s head is important; we need everyone to be a part of the team and understand why it matters. For example*,* why should everyone be able to carry out a NEWS [National Early Warning Score]; because it is related to patient safety and the quality of our work. It does not matter what decisions managers make if people do not understand the “why” and “what”; they will non implement it* (P5).

Several participants said that their QI work focused directly on enhancing the clinical knowledge of healthcare professionals in the workplace. Clinical knowledge, they noted, must be emphasized continuously. The participants also indicated that, through the QIC, they had discovered a new approach to work in this important area. They noted that the goal of this approach was to ensure that their QI work had a lasting impact, and by implementing it across multiple units within the municipality in which they worked, they aimed to increase quality and patient safety. This emphasis on the task of improving the knowledge of healthcare professionals was an element that several participants felt had been lacking and highlighted by one participant:

*Just focusing on professional development*,* that’s something that I have missed. Finally*,* I say* (P5).

Many participants highlighted the belief that their participation in the QIC had increased their knowledge regarding QI work.

### *Challenges in efforts to maintain participants’ motivation over time*

The participants discussed the importance of QI work and acknowledged the support provided by both managers and colleagues. They also described various influences on motivation during the long-lasting processes associated with QI work and highlighted the importance of experiencing a sense of progress and usefulness even when this process took time to complete. According to one participant:

*It must be perceived as highly useful because no additional time has been allocated*,* and you have not been given any extra time. Moreover*,* it must fit into the existing workload. If it is not perceived as useful*,* it becomes burdensome* (P1).

The participants said it was crucial to remain motivated and continue performing QI work even after the QIC ended. One participant made the following comment:

*Having a focus on the continuous and not putting it aside once we finish the project*,* it must continue to live; it needs to be a system*,* and routines must be put in place to follow up on it further ahead* (P2).

Some participants highlighted the risk of reverting to previous practices in the absence of such follow-up. Several participants claimed that significant instability among employees could make it challenging for the manager to motivate them to engage in QI work:

*There is high turnover here*,* with people being sick*,* on leave*,* in temporary positions and moving around. They are pursuing education*,* and there are many part-time positions* (P10).

Several participants claimed that it was important for the manager to follow up on QI work after the completion of the QIC.

## Discussion

This study of the experiences of employees in municipal healthcare services in Norway with participation in a digital QIC revealed that the QIC sessions and the process guidance provided between sessions were important for maintaining participants’ learning in the community with others. Furthermore, the participants managed to remain in the preparation phase longer when they used the Quality Improvement Model, and leadership anchoring and involvement were essential to encourage employees to participate in QI work and implement the changes required by such work.

The participants in the present study described the QIC sessions and the sharing of experience as crucial to their efforts to learn from others and maintain the momentum of their QI work. This exchange of experiences also facilitated progress in their own work, as all the teams were required to present their QI work as part of the QIC sessions. A systematic review that focused on how QIC can promote better outcomes, revealed that QIC can positively impact healthcare professionals’ knowledge, attitudes and problem-solving abilities [7]. OIC can also be regarded as an opportunity to promote methodology for QI work [9]. According to Vygotsky [28], the establishment of a supportive learning environment is important for learning processes. Conducting QIC entirely digitally has rarely been explored, and the participants in this study provided mixed experiences. Some participants found the digital sessions more difficult to follow, and initially preferred a physical lecturer, whereas others noted that unfamiliarity with other participants raised the threshold for speaking up. Rohweder et al. [29] found that independent practitioners who participate in a virtual QIC were satisfied and resulted in high participation. Conducting QIC digital may have limited learning outcomes for some participants in our study, yet they enable broader participation by reducing time and travel costs, which is an important benefit in strained municipal economies. Digital gatherings may also facilitate participation from a wider geographical area, thereby ensuring the dissemination of important knowledge. This suggests that participation in a facilitated digital QIC can enhance employee engagement, support learning, and help maintain momentum in QI work.

The participants in our study said that the process guidance provided between the QIC sessions was necessary for them to understand the subsequent phase and to ensure that they made progress in their QI work. They indicated that the supervisors were well prepared, listened to their challenges, and provided positive feedback that helped ensure that team motivation remained high. Zamboni et al. [7] and Carstensen et al. [9] highlighted the importance of providing sufficient and appropriate external support during QI work with the goal of ensuring the effective functioning of QIC teams and the success of organizational change. In our study, local nurses holding a master’s degree employed in USHT were trained to act as supervisors in QIC. This plays a crucial role in ensuring follow-up after the conclusion of the QIC. Process guidance by supervisors should be rooted in a holistic approach that emphasizes change processes that unfold over time rather than solely focusing on specific topics pertaining to individual projects [9, 30]. Owing to their participation in the QIC and the process guidance that they received between the sessions, the participants in our study acquired experience and thus received the support that they viewed as necessary to support their QI work. The Institute for Healthcare Improvement [23] seeks to improve health care by supporting change, and they believe that collaborative learning through the QIC is one way to achieve this. Vygotsky [28] uses the term “scaffolding” to describe the facilitation and support of others’ learning. The present study suggests that guidance from skilled supervisors during participation in QIC provides essential support that enhances participants learning processes.

The participants in the present study said that the use of the Quality Improvement Model enabled them to remain in the preparation phase longer, thus refining their QI work and making their projects more robust. Coury et al. [31] reported similar results, using a QI model made it easier for participants to remain in the initial phase and helped them focus on the necessary task of planning and organization prior to implementation. According to Carstensen et al. [8] the systematic use of a QI model is a tool to focus an improvement project, both on what one wants to improve and on how to do it. However, multiple studies have demonstrated that many QI projects do not follow a QI model consequently, these projects feature limitations with respect to planning, as the theoretical rationales underlying such projects are weak [20, 32]. Avby [33] emphasizes the importance of a thorough planning process in QI work and proposes a seven-step framework for innovative work that bears some resemblance to the Quality Improvement Model. Avby’s [33] framework explorative phase helps individuals and teams linger lightly longer in the problem-formulation phase, thereby allowing them to carefully understand the complexity and reducing the risk of a predetermined solution. Harrison et al. [34] name this planning phase the work before the work. Some teams in our study progressed too quickly in their QI work and thus found it necessary to take a step back to clarify the problems and scale down their projects to make them feasible. This highlights the importance of the new preparation phase in the Quality Improvement Model, and it addresses the deficiency that Reed and Card [17] have identified in the PDSA cycle. Even though it was challenging for the staff to devote considerable time to the preparation phase, they experienced it as beneficial. Overall, the present study emphasizes the importance of ensuring that QI projects are preceded by thorough preparatory work, which builds confidence that these initiatives will lead to real improvements.

The participants in the present study highlighted the importance of ensuring that all healthcare professionals in the QIC understand the Quality Improvement Model and recognize the importance of engaging in QI work. Multiple studies revealed that the introduction of the PDSA cycle requires a fundamental change in how QI team members think and approach a QI project. Such fundamental changes require thorough training in the methodology used in this context prior to the implementation of the QI project [18, 35]. Training in the use of QI models in QICs is important and can promote collaborative learning [11, 36].

Several participants in the present study emphasized that leadership anchoring and involvement are essential with respect to encouraging employees to participate in QI work and implement the changes required by QI work. The participants indicated that without such leadership anchoring, the task of implementing QI could be challenging, and the process of sustaining these changes following the QIC could be difficult. At the same time, some teams found it challenging to secure leadership anchoring for their projects. According to Devi et al. [32] managers play a crucial role by leading QI work in their care homes. These authors found that leaders propose ideas for improvement, organize QIC meetings, and ensure the implementation of improvement interventions in their care homes. Other studies have shown that enganging managers are crucial for the success of QIC and QI work as they play a key role in planning and implementation. Leadership involvement, supported by a culture that prioritizes QI and allocates resources, is pivotal to QICs [7, 37]. Sjølie et al. [38] highlighted that establishing routines for QI work is a central task for first-line nurse managers, however it is a demanding task since their days involve frequent interruptions.

The findings of this study emphasise the importance of enabling all employees to participate in QI work and understanding the purpose of QI work and the critical role it plays in patient safety; otherwise, implementing corresponding changes would be difficult. Gadolin and Andersson [39] examined various conditions that influence the ways in which employees engage in QI work and found that, to engage healthcare professionals in QI work, ensuring that employees perceive this work as important is crucial, otherwise they are unwilling to participate in this process. Orvik et al. [40] revealed that collaboration works well at the top level of organizations but can be more challenging among employees who are directly involved in QI processes. The findings of our study highlight the importance of involving both leaders and employees in QI projects. QI work increases user satisfaction and leads to more efficient work processes when applied in a structured manner and anchored in leadership. Learning and continuous feedback facilitate the long-term improvement and quality development of employees’ participation in quality improvement projects to higher degree of job satisfaction and lower absenteeism [1, 10].

QI work in the context of healthcare is embedded in complex systems, thus making it crucial for managers to focus on structures such as staffing levels, routines, equipment, and central guidelines as well as the organizational culture, including norms and values that are important to consider both in QI work and in the process of implementing the changes required as a result of such QI work [14].

### Strengths and limitations

One strength of this study lies in the fact that it investigates the process of working with QI on the basis the Quality Improvement Model while supplementing a digital QIC with process guidance. Previous studies on this topic seems to be lacking. This insight will highlight how the model functions in practice and its contribution to strengthening QI work. At the same time, it captures the experiences of employees in the municipal healthcare services participating in a digital QIC, providing knowledge that may be significant for the design of future QICs. This may hold relevance in an international context.

To ensure trustworthiness, credibility, transferability, dependability and confirmability were applied throughout the study [41]. The purposive sampling of employees with experience in participation in the QIC and use of the Quality Improvement Model contributed to strengthening credibility. The small number of participants in each group, combined with the fact that only ten participants from the QICs were included and only one without higher education, is a notable limitation. This may lead to less variation in experiences and perspective, which can reduce the breath of the findings, and it may also affect the transferability of the results as well as data saturation. However, the data collected from each focus group were rich and varied. The variation observed in participants’ backgrounds may have contributed to this situation [21], alongside the possibility that smaller groups may make it easier for participants to engage in discussion [42]. The fact that all the participants were women may also have affected both the transferability, however, they mirror the composition of employees in the municipal health services, where woman is overrepresented. However, the sample size was considered sufficient. To ensure transferability a clear description of the context, data collection and analysis were provided. A clear description of the research process also contributes to strengthening the study. Reflexivity was further ensured through researchers’ awareness of preunderstanding and ongoing reflection among the researchers during the study. The use of the Consolidate Criteria for Reporting Qualitative Research (COREQ) checklist ensured that this study was conducted transparently [43].

## Conclusion

The present study reveals that the Quality Improvement Model is suitable for QI work and QICs in municipal healthcare service, and the extended first phase of the model was evaluated by the participants with the goals of identifying their actual needs and clarifying the aims of their QI projects. The model was new to the participants, but they found it to be useful for both minor and major QI projects. This study highlights the importance of providing process guidance between digital QIC sessions with respect to the progress of QI work. To ensure effective QI work, leadership anchoring and the involvement of managers and employees who are familiar with both QI work and the Quality Improvement Model were identified as crucial. Managers play a crucial role in the task of following up on QI work after projects are completed, thereby ensuring that changes are sustained and implemented successfully. The findings of this study reveal that it can be beneficial for healthcare practice to use the Quality Improvement Model in subsequent QI work, as the newly included phase (i.e., the preparation phase) helps strengthen QI work. Further researchers should examine the effects of using QICs and the Quality Improvement Model in the context of QI work in municipal healthcare service and how the model works outside Norway.

## Data Availability

Owing to issues pertaining to participant confidentiality, the original audio and transcripts of the focus group discussions are not publicly available.

## Abbreviations

QIC: Quality improvement collaborative
QI: Quality improvement
QI work: Quality improvement work
PDSA: Plan‒Do‒Study‒Act
